# Involving seldom-heard groups in a PPI process to inform the design of a proposed trial on the use of probiotics to prevent preterm birth: a case study

**DOI:** 10.1186/s40900-017-0061-3

**Published:** 2017-05-25

**Authors:** Juliet Rayment, Rosemary Lanlehin, Christine McCourt, Shahid M. Husain

**Affiliations:** 1 School of Health Sciences, City, University of London, 1 Myddelton Street, London, EC1R 1UW UK; 2 Centre for Genomics and Child Health, Blizard Institute, Barts and the London School of Medicine and Dentistry, Queen Mary University of London, 4 Newark Street, London, E1 2AT UK

**Keywords:** Clinical trial acceptability, Feasibility studies, Patient and public involvement, Advisory groups, Public understanding of clinical trials, Women’s health

## Abstract

**Plain English summary:**

When designing clinical trials it is important to involve members of the public, who can provide a view on what may encourage or prevent people participating and on what matters to them. This is known as Public and Patient Involvement (PPI). People from minority ethnic groups are often less likely to take part in clinical trials, but it is important to ensure they are able to participate fully so that health research and its findings are relevant to a wide population. We are preparing to conduct a randomised controlled trial (RCT) to test whether taking probiotic capsules can play a role in preventing preterm birth. Women from some minority ethnic groups, for example women from West Africa, and those who are from low-income groups are more likely to suffer preterm births. Preterm birth can lead to extra costs to health services and psychosocial costs for families. In this article we describe how we engaged women in discussion about the design of the planned trial, and how we aim to use our findings to ensure the trial is workable and beneficial to women, as well as to further engage service users in the future development of the trial. Four socially and ethnically diverse groups of women in East London took part in discussions about the trial and contributed their ideas and concerns. These discussions have helped to inform and improve the design of a small practice or ‘pilot’ trial to test the recruitment in a ‘real life’ setting, as well as encourage further PPI involvement for the future full-scale trial.

**Abstract:**

**Background**

Patient and public involvement (PPI) is an important tool in approaching research challenges. However, involvement of socially and ethnically diverse populations remains limited and practitioners need effective methods of involving a broad section of the population in planning and designing research.

**Methods**

In preparation for the development of a pilot randomised controlled trial (RCT) on the use of probiotics to prevent preterm birth, we conducted a public consultation exercise in a socially disadvantaged and ethnically diverse community. The consultation aimed to meet and engage local service users in considering the acceptability of the proposed protocol, and to encourage their participation in future and ongoing patient and public involvement activities. Four discussion groups were held in the community with mothers of young children within the proposed trial region, using an inclusive approach that incorporated a modified version of the Nominal Group Technique (NGT). Bringing the consultation to the community supported the involvement of often seldom-heard participants, such as those from minority ethnic groups.

**Results**

The women involved expressed a number of concerns about the proposed protocol, including adherence to the probiotic supplement regimen and randomisation. The proposal for the RCT in itself was perceived as confirmation that probiotic supplements had potentially beneficial effects, but also that they had potentially harmful side-effects. The complexity of the women’s responses provided greater insights into the challenges of even quite simple trial designs and enabled the research team to take these concerns into account while planning the pilot trial.

**Conclusions**

The use of the NGT method allowed for a consultation of a population traditionally less likely to participate in medical research. A carefully facilitated PPI exercise can allow members to express unanticipated concerns that may not have been elicited by a survey method. Findings from such exercises can be utilised to improve clinical trial design, provide insight into the feasibility of trials, and enable engagement of often excluded population groups.

## Background

There is growing interest in using PPI in clinical trials, from priority setting [1, 2] through to involvement with study design and conduct [3]. Priority-setting ensures that research is responsive to patients’ concerns [4]; and involvement with study design means that potential challenges for individual research projects can be identified and addressed at an early stage [5]. INVOLVE defines public involvement in research as ‘research being carried out ‘with’ or ‘by’ members of the public rather than ‘to’, ‘about’ or ‘for’ them. This includes, for example, working with research funders to prioritise research, offering advice as members of a project steering group, commenting on and developing research materials and undertaking interviews with research participants’ [6]. This article describes a public consultation exercise, used as a first step towards influencing the design of a clinical trial, and with the aim to establish continued involvement throughout the trial.

To understand research needs and challenges, PPI has to engage people who are able to offer valid perspectives from the study population. Widening inclusion has sound scientific value [4]. Population groups might have different perspectives informed by, for example, socio-economic status, ethnicity, health status, or gender, which might impact on participants’ recruitment and retention rates in clinical trials [4, 7, 8]. PPI can help to identify possible problems with recruitment and retention at an early stage, by developing a better understanding of any barriers that might exist [9–12]. Being inclusive of people from diverse backgrounds in consultation work is also a matter of health equity. Barriers to participation that go beyond the more often cited structural barriers within healthcare (such as time and staffing), or individual choice (such as preferences for certain treatments, or concerns about efficacy of a new treatment), may be highlighted [7, 8]. Despite positive changes in policy and practice that no longer automatically exclude people from ethnic minorities in medical research, people from minority ethnic groups may be systematically excluded from participation in clinical trials due to a number of indirect factors, such as mistrust, inappropriate exclusion criteria, access difficulties, interpretation and translation costs, socio-cultural barriers, and cultural myths [13, 14].

Early PPI in the development of a trial can yield valuable findings [8, 9], but consultations often occur later in the development process, such as during a pilot trial. Boote et al’s review of PPI in trials identified that even in trials with involvement at the design stage this was mainly in designing materials and leaflets for patients and agreeing outcome measures and tools, or in monitoring the process, rather than at an earlier stage of the design [4]. In contrast, Edwards et al’s study showed that by involving families early in the design of a trial for children with cerebral palsy, the research team was able to identify basic design approaches and wider outcome measures that could help improve the acceptability and take-up of the planned trial [5]. This study was, however, based on interviews with parents involved in an active user group and such approaches could themselves be limited in addressing issues relevant to a diverse population.

Building on current knowledge about the benefits of PPI, the project reported here aimed to actively involve mothers of young children in considering the feasibility and design of a proposed RCT: “PrePro - Preventing preterm birth with probiotics”. The intended study population has a higher than national average rate of preterm birth and marked diversity of socio-economic status and ethnicity [15]. The target population is often considered as “hard to reach”, both in terms of geographical location and in terms of being engaged with research. We acknowledged the benefits of carrying out such consultations in the community, to avoid difficulties with travel to a central location [16]. The proposed full-scale trial is a multicentre, placebo-controlled, double-blind RCT examining the effects of daily probiotics from early pregnancy until the end of gestation on the vaginal microbiological flora and risk of preterm birth [17–19]. To determine a clinically significant effect, if any, on the risk of preterm birth, it is anticipated that 10,000 participants may be required in the RCT. Therefore, a pilot trial on 366 participants is currently underway in order to assess acceptability and feasibility prior to conducting the full-scale trial. In preparation for the pilot trial, we considered it important to consult with representative service users in order to get any insights on the trial design.

The objectives of this PPI project were:

- to convene four discussion groups involving mothers of young children within the intended trial region. i.e. East London;
- to ensure inclusion and involvement of women across the diverse community;
- to actively involve women in discussion about the trial design and seek routes for ongoing engagement;
- to learn about the potential barriers and facilitators to carrying out the trial; and
- to consider the acceptability of the intervention: one probiotic or placebo capsule per day from recruitment to the end of pregnancy and three self-administered vaginal swabs at routine antenatal appointments.

## Methods

### PPI representatives and settings

Four mothers’ group discussions were held. Three were with mothers of babies under 1 year of age, between July and September 2013. The mothers were contacted through Children’s Centres in three boroughs of East London (Hackney, Waltham Forest and Newham). As women from some minority ethnic communities (particularly those of West African origin) are disproportionately affected by preterm birth in the UK, and are also less likely than White British women to participate in clinical research [8, 15], we specifically sought involvement of West African women through a church in the borough of Tower Hamlets, where the fourth discussion group was held. The group of women taking part were acting as advisors to the researchers and had no direct contact with research participants, therefore ethical approval from the National Research Ethics Service was not required, as outlined in the guidelines from INVOLVE and the National Patient Safety Agency [6, 20]. Taking part in the discussions was voluntary and all discussion group members gave informed consent for their contributions to be recorded in the written notes.

A total of 35 women took part in the group discussions. One group included a female practising midwife and a male GP, who were members of the Church group and interested in the topic. The 35 women were aged between 27 and 43 years old, and were from a variety of ethnic backgrounds including: White British (*n* = 5), White Other (*n* = 3), Indian (*n* = 1), Pakistani (*n* = 4), East African (*n* = 1), West African (*n* = 18) and Black Caribbean (*n* = 3). Whilst we were unable to be fully representative of the local population due to the small numbers of women who took part, the distribution is reflective of the local communities in Tower Hamlets, Waltham Forest and Newham, which have 69, 48 and 83% of their population respectively from Black and Minority Ethnic groups. We did not collect any indicators of socio-economic status, such as income or educational level, but the Local Authorities in which this research took place are all within the top 40 most deprived in England.^1^

As with all aspects of research, engaging diverse groups of people can at times be challenging and individuals’ willingness to engage with PPI may mirror their willingness, or otherwise, to engage with clinical research, health services or state institutions. A number of key strategies were deployed in order to minimise the barriers to attending the discussion groups. The three Children’s Centre groups existed previously to the project and the project team ensured that the discussions were planned in a time and place when the women were already meeting. Our previous experience of engaging women from communities across Britain [1, 21, 22] had shown that it was often impractical to ask women with small babies to attend an extra group meeting at a particular time of day. In contrast, visiting existing baby drop-in groups was significantly more successful as women were used to attending these groups at regular intervals and were also in a setting where they felt comfortable.

### The Nominal Group Technique – standard and modified

The four groups were facilitated using a modified version of principles drawn from Nominal Group Technique (NGT), which allows collective ‘brainstorming’ and group decision-making [23, 24]. The principles were used to provide an initial structure for the meetings, but were applied in a very flexible fashion in response to the group on the day. The aim of NGT is to give individuals an equal opportunity to contribute to collective decision-making and to attempt to mitigate against group dynamics based on personality and the social dynamics of power. When using NGT, the facilitator allows everyone in the group time alone to consider and record as many responses as possible to a question. The responses are then collected by the facilitator and shared with the group or read out by group members. No criticism, comment or judgement is made on the responses by the facilitator, but they may offer clarification or responses to specific questions. The group then engages in an open and detailed facilitated discussion of the issues raised. Finally, group members vote on which responses they feel are important or useful, often using a ranking approach.

We knew from previous research with the Bangladeshi community in East London that women in that community preferred to engage with healthcare professionals through face-to-face discussions rather than written materials [21] and this kind of community intelligence was crucial to guiding our approach for this project. In addition, group discussions allow the conversation to flow, without a specific agenda. Whilst we had pre-existing ideas about what some barriers or facilitators to trial participation might be, these did not set the topic of the conversations. Convening group discussions like this better supported service users to engage with the design of the trial and understand what was being asked of them. Due to the nature of this method, we did not know who would be attending the groups in advance. This meant we could not plan interpretation for women who may have come and who spoke no English. However, with slow and careful facilitation and small group working, women with limited spoken or written English were supported to participate by their peers. Some informal interpretation between participants was common during the sessions, and volunteer ‘scribes’ within pairs or small groups ensured that everyone’s ideas were included in the written activity.

Whilst the discussions used a number of tools from NGT, our strategy differed in a number of ways from the standard NGT model. An A1-sized flow chart representing the planned trial protocol was used to help explain the recruitment, randomisation and intervention involved in the trial, in a visual manner. The proposed protocol was explained verbally to each group by the researcher. Each group was asked to imagine that they were being invited to join the trial at their routine antenatal dating scan appointment and consider, hypothetically, what questions or concerns they might have about participating. Each group was told that this information would be used to make adjustments to the procedures for the trial or the information given to future trial participants, to help ensure that the trial is as ‘user-friendly’ as possible for those who are asked to participate. We gave reassurance that contributing to the PPI exercise did not mean that the women present were being asked to participate in the trial, nor would they necessarily be in the future. However, we did seek each participant’s interest in continuing their involvement as a PPI representative.

The women’s concerns and questions were written onto individual sticky notes by the women in the group or by the researcher and placed onto the flowchart at the point in the process to which they referred. We did not attempt to actively answer or address women’s concerns and questions during the activity; these were simply recorded, but some additional clarification of the trial protocol was given during the discussion if needed. We did not require the women to rank or vote on the responses as in standard NGT; all ideas were considered of equal value to developing the protocol. By working with the discussion group to address the protocol step-by-step, the women in the groups contributed to the analysis of their own contributions. The women sorted the concerns and questions according to which part of the protocol they addressed (e.g. recruitment, randomisation, vaginal swab, probiotic capsule regimen, qualitative interviews). Following the discussion groups, all queries were collected and transcribed for future reference.

### Additional PPI activity

Complementary to the NGT session, during the pilot trial we involved the women’s health research advisory group “Katie’s Team”, which is based in East London and is made up of patient and public representatives. The women who participate in Katie’s Team reflect the ethnic make-up of the area, being 60% of South Asian origin, 5% of West African origin and 35% of a number of other ethnicities [25]. This advisory group consults on numerous research projects and clinical trials within women’s health, and so their involvement with PrePro brings great value to our discussions on trial design, feasibility and progress.

Women who were interested in being involved in the ongoing development of the trial were asked to provide their contact details. These women (*n* = 9) were later re-contacted during the pilot trial and invited to contribute further to the project’s development. These contributions included being invited to join Katie’s Team and to volunteer as PPI representatives on the trial steering group.

## Results

A key aim of the public consultation exercise was to assess the acceptability of the protocol of a daily probiotic capsule and three self-collected vaginal swabs. Whilst a quantitative research approach using a questionnaire would have enabled us to evaluate the views of a larger number of women in the trial region, there were a number of concerns with using questionnaires. Firstly, they rely on good levels of literacy in English, which could not be guaranteed in the target geographical region. Secondly, they rely on volunteers completing them and returning them by post, or online, which has been linked to a very poor response rate amongst more deprived populations [21]. Thirdly, the development of a survey requires some initial hypotheses or assumptions about the problems women may find during the trial, thereby restricting the possible findings within the boundaries of those hypotheses.

We were able to involve a diverse sample of women by making contact with existing community-based groups: women attending Children’s Centre baby drop-in groups and members of a local church, rather than requiring women to attend a focus group in an unfamiliar venue. Children’s Centres have traditionally provided services for women in lower socio-economic groups. Whilst their catchment has diversified in recent years, many are still used by a more socially disadvantaged and diverse sector of the population. Although Children’s Centre groups do not necessarily include women who are significantly socially isolated, we could consult women whose voices are seldom-heard in PPI. The church group was contacted specifically to ensure that we involved women of West African origin, as they are disproportionately affected by preterm birth. The flexibility of the approach allowed women to bring up any concerns or questions they had. Furthermore, discussions developed from each individual’s initial suggestions, which meant that others were encouraged to think differently about their own potential participation, and to agree or to refute others’ suggestions. The women became more actively engaged in the process of thinking about and planning the trial.

### Adherence and randomisation

One of the biggest findings was that many women were concerned about the practicalities of taking part in the trial and did not have confidence that they would remember to take the capsules in accordance with the protocol, as pregnancy was already a demanding time in their lives. Women were also often concerned about being randomised, particularly to the placebo arm of the trial. This concern could be expected, but it introduces a particular threat to the success of this trial, which would test products that are freely available to participants over the counter. Rather than participating in the trial to gain access to otherwise unavailable medications, which may be the case in many drug trials, the women taking part in this exercise were more concerned that participating in this trial could deny their access to potentially beneficial, widely available, probiotic supplements. Some participants suggested that the possibility of receiving a placebo after consenting to participate might encourage them to take ‘over the counter’ probiotics to supplement the trial.

### Women’s perceptions

The most novel insight emerging from these discussions was in relation to women’s perceptions of the safety of the probiotics themselves. It appeared that the process of explaining a trial to potential participants may heighten awareness of both the potential benefits and the potential risks of an intervention. All the women had heard of probiotics or ‘friendly bacteria’, however, many women were hesitant about taking probiotic capsules during pregnancy as part of a clinical trial. A large number of women felt that it was confusing to be asked to take tablets in a trial when they were otherwise discouraged from taking tablets during pregnancy. One woman was concerned that bacteria were ‘live’ and could therefore be damaging. Many women believed that all tablets had some side effects and were worried about the ‘inevitable’ side effects of taking probiotic capsules. Although some women said they had used or continued to use probiotics as a dietary supplement, they still expressed concern about their safety within the context of the trial and in a ‘tablet’ form. Interestingly, the exact same concerns of taking a tablet being perceived as ‘medicine’, and therefore potentially harmful, were also expressed during consultation with members from ‘Katie’s Team’.

## Discussion

This approach to facilitating discussion around the trial was found to be effective in meeting our PPI objectives, particularly in terms of involving a more diverse range of women in the community in discussing and commenting on the plans and it provided useful information to inform the pilot trial design and planning. Women raised concerns and questions that were consistent across each of the four groups, highlighting issues that we had anticipated and some that we had not.

Whilst both concerns regarding adherence to the protocol and randomisation could be easily anticipated, the group discussions brought up some further findings that were less readily expected. Although we had expected women to be more concerned by the more invasive part of the participation requirements, taking three self-administered swabs was far less of a concern to women than taking daily capsules. Vaginal swabs were perceived as common in pregnancy and all but two of the group members said they would be happy to carry out a vaginal swab on themselves. Additionally, timing the swabs to coincide with antenatal appointments would minimise demands on participants. One of the two women who was not willing to carry out self-administered swabs had been advised by her GP not to have unnecessary swabs during pregnancy and was worried about their safety. The other woman was more comfortable with a health professional taking a swab than it being self-administered.

Many women’s perception of the probiotic was transformed from ‘dietary supplement’ to ‘drug’ by the proposed clinical trial itself. It is possible that probiotics administered in any form other than a capsule (for example, a change in diet or a probiotic drink) may have been more acceptable to potential participants. The existence of the trial was seen as confirmation that probiotics had potentially beneficial effects on preterm birth, but also that they had potentially harmful side-effects. The women appeared to simultaneously hold two, conflicting positions: a desire for the benefits of the probiotics and concern that they were unsafe. The ability of humans to maintain and act on such complex, apparently contradictory views may be underestimated by researchers, who often draw on a more linear and rationalistic model of human decision-making.

## Conclusions

The approach described here was effective in reaching and engaging women from diverse backgrounds, enabling involvement of a range of women who might be approached for participation in a pregnancy-related trial. The collaborative group discussions enabled an open, exploratory approach, which did not confine the findings to researchers’ existing hypotheses, but instead enabled fresh issues to emerge from the women’s perspectives. It gave us greater insight into the considerations to be addressed in designing and implementing a trial on this topic and in this typically ethnically diverse and socially deprived region. This type of involvement can complement some of the more conventional PPI strategies, such as inviting individual PPI representatives to research team meetings or steering committees, where the discussion is tailored towards and paced for the needs of the researchers, rather than the service users. Going to where community members are rather than expecting participants to come to where the researchers are is a simple but under-utilised approach to engaging seldom-heard groups. This accessible approach also engaged the women’s interest and enthusiasm for research, reflected in the number of women (*n* = 9) from a range of ethnic backgrounds who volunteered to be contacted and maintain an ongoing involvement as user representatives. The group discussions enabled the research team to identify clear lessons to inform the design of a pilot trial, which is ongoing. The pilot trial has included a qualitative evaluation of the process of recruitment, which is further exploring the ideas generated by this consultation exercise and testing their impact on ‘real life’ trial recruitment. In combination with the public consultation through Katie’s Team, the findings from this evaluation informed the development of the pilot trial protocol, and will continue to inform the development of the future definitive trial. Trial researchers facing similar challenges could also consider some of changes that we made in response to this exercise:

- We ensured that the information presented to participants at recruitment clearly explained the concept of randomisation; the nature of the specific probiotic supplements and the fact that they are not available over the counter; and their potential effects during pregnancy.
- When in discussion with participants, we consistently described the probiotic in terms of a ‘food supplement’ rather than a ‘medicine’.
- As there were no concerns with collecting vaginal swab samples, we ensured the protocol allowed samples to be self-collected as well as by a healthcare professional.
- Each sample collection was timed with the regular antenatal appointments, to enable participants to remain compliant.
- For the definitive trial, we may consider providing the probiotic supplement in another format other than a capsule: as a drink or a powder for example.

Continued involvement of PPI representatives, as described in this study, will be an invaluable component in considering the feasibility of progressing to a large-scale trial and could provide a route towards engagement of a more diverse range of people in such PPI roles. However, research teams need to be mindful that not all members of the public will feel able (for practical reasons such as time) or confident to take on a formal PPI representative role. Therefore, ongoing encouragement and support for participation and consideration of how PPI representatives can be involved in groups such as Katie’s Team and in trial steering groups also requires detailed attention.

## Abbreviations

NGT: Nominal Group Technique
PPI: Patient and public involvement
PrePro: Preventing preterm births with probiotics
RCT: Randomised controlled trial

## Footnotes

1 The three boroughs are ranked 11^th^, 24^th^ and 36^th^ most deprived by average Index of Multiple Deprivation score (2015). Data available from the Office of National Statistics at: https://www.gov.uk/government/statistics/english-indices-of-deprivation-2015.
